# Plasma Pharmacokinetics and Tissue Distribution of Doxorubicin in Rats following Treatment with Astragali Radix

**DOI:** 10.3390/ph15091104

**Published:** 2022-09-04

**Authors:** Yin Huang, Fang Yang, Linling Guo, Yan Xu, Xiaxia Yu, Zunjian Zhang, Yuxin Zhang

**Affiliations:** 1 China Pharmaceutical University Nanjing Drum Tower Hospital, Nanjing 210009, China; 2 Key Laboratory of Drug Quality Control and Pharmacovigilance, China Pharmaceutical University, Ministry of Education, Nanjing 210009, China; 3 Department of Pharmacy, Affiliated Zhongda Hospital, School of Medicine, Southeast University, Nanjing 210009, China

**Keywords:** Adriamycin, drug disposition, combination therapy, LC-MS/MS, network

## Abstract

Doxorubicin (DOX) is an essential component in chemotherapy, and Astragali Radix (AR) is a widely used tonic herbal medicine. The combination of DOX and AR offers widespread, well-documented advantages in treating cancer, e.g., reducing the risk of adverse effects. This study mainly aims to uncover the impact of AR on DOX disposition in vivo. Rats received a single intravenous dose of 5 mg/kg DOX following a single-dose co-treatment or multiple-dose pre-treatment of AR (10 g/kg × 1 or × 10). The concentrations of DOX in rat plasma and six tissues, including heart, liver, lung, kidney, spleen, and skeletal muscle, were determined by a fully validated LC-MS/MS method. A network-based approach was further employed to quantify the relationships between enzymes that metabolize and transport DOX and the targets of nine representative AR components in the human protein–protein interactome. We found that short-term (≤10 d) AR administration was ineffective in changing the plasma pharmacokinetics of DOX in terms of the area under the concentration–time curve (AUC, 1303.35 ± 271.74 μg/L*h versus 1208.74 ± 145.35 μg/L*h, *p* > 0.46), peak concentrations (C_max_, 1351.21 ± 364.86 μg/L versus 1411.01 ± 368.38 μg/L, *p* > 0.78), and half-life (t_1/2_, 31.79 ± 5.12 h versus 32.05 ± 6.95 h, *p* > 0.94), etc. Compared to the isotype control group, DOX concentrations in six tissues slightly decreased under AR pre-administration but only showed statistical significance (*p* < 0.05) in the liver. Using network analysis, we showed that five of the nine representative AR components were not localized to the vicinity of the DOX disposition-associated module. These findings suggest that AR may mitigate DOX-induced toxicity by affecting drug targets rather than drug disposition.

## 1. Introduction

Doxorubicin (DOX), also known as Adriamycin, is widely used in clinics for the treatment of many different types of cancer, such as acute myeloid leukemia and breast cancer [1]. DOX stops or slows the growth of cancer cells by damaging DNA and chromatin, but it also kills normal cells, resulting in a series of side effects such as arrhythmia and bruising [2,3]. The clinical application of DOX is limited by its severe side effects. For example, to prevent cardiotoxicity, elderly cancer patients with heart problems are often excluded from chemotherapy regimens containing DOX. Combination therapy using multiple drugs to improve clinical outcomes offers a strategy to mitigate DOX-induced toxicity with efficacy [4,5].

Astragali Radix (AR) is a common, traditional Chinese medicine that has long been used as an anti-oxidative and anti-inflammatory herbal prescription for multiple complex diseases, from diabetes to asthma and cancer [6]. Evidence suggests that AR has a therapeutic potential to alleviate the DOX-induced toxicity of the heart, liver, and kidney [7,8]. While cell death is a unifying feature of DOX-induced toxicity, it is not clear how AR could simultaneously interfere with the death programs in different organs. The protective effects of AR probably involve pleiotropic mechanisms, including regulation of enzymes and metabolites that fuel energy metabolism, inhibition of pro-inflammatory cytokines, and suppression of oxidative stress [9,10,11].

DOX administered as a conventional injection is widely distributed in the plasma and tissues but can hardly cross the blood–brain barrier [12]. There are three main metabolic routes of DOX in mammals: one-electron reduction, two-electron reduction, and deglycosylation [13]. Previous studies have shown that lowering the level of DOX in normal organs is beneficial in reducing toxicity. For example, the liposomal formulation of DOX, which reduces myocardial drug accumulation, has provided a significant reduction in the risk of cardiotoxicity [14,15]. When DOX is combined with dandelion to treat breast cancer, dandelion can reduce the intracellular accumulation of DOX by activating the drug efflux transporter P-glycoprotein, thereby reducing DOX-induced cardiotoxicity [16]. It is possible that AR protective effects involve a similar mechanism. However, there has been no report on the effect of AR on the pharmacokinetics and tissue distribution of DOX.

Here, we hypothesized that, in addition to acting on DOX targets, AR might mitigate the side effects by reducing DOX exposure in vivo. To test this hypothesis, we developed and validated a simple, specific, and sensitive liquid chromatography–tandem mass spectrometry (LC-MS/MS) method for the determination of DOX in rat plasma and six tissues (heart, liver, lung, kidney, spleen, and skeletal muscle), allowing us to analyze the effects of AR co-treatment and pre-treatment on DOX disposition. Moreover, enzymes that metabolize and transport DOX are not randomly scattered in the human protein–protein interactome (PPI), but tend to cluster in the same neighborhood, known as the DOX disposition-associated module [17]. The targets (usually proteins) of AR components do this as well. If AR does change the in vivo metabolism or efflux of DOX, some AR components must be localized to the vicinity of the module related to DOX disposition [18]. Therefore, we further proposed a network-based measure that helped us quantify the topological relationship between nine representative AR components and DOX disposition, offering a novel approach to understanding the potential mechanism.

## 2. Results

### 2.1. Method Validation

The representative extracted ion chromatograms of DOX and IS in rat plasma and tissues are illustrated in Supplementary Figure S1. DOX showed good linearity (r^2^ > 0.995) in all biological samples with broad dynamic quantification ranges, from two to three orders of magnitude (Table 1). Intra-day and inter-day accuracy and precision were appropriate for all determinations within the linear range. Precision, measured as RSD, was below 10.6% and accuracy ranged from 89.15% to 104.26% (Table 2). Recovery of DOX was over 77.32% in plasma and most tissues, except for the spleen, which ranged from 66.34% to 72.23% (Supplementary Table S1). The possible matrix effects were also studied, and no significant suppression or enhancement was observed (Supplementary Table S1). Moreover, DOX was found to be stable in all biological matrices under different conditions, including autosampler, short- and long-term storage, and three freeze-thaw cycles (Supplementary Table S2). These results demonstrate that the LC-MS/MS method is sensitive and accurate for the reliable quantification of DOX present in rat plasma and six tissues.

### 2.2. Effects of Astragali Radix on Doxorubicin Disposition

**Figure 1:**
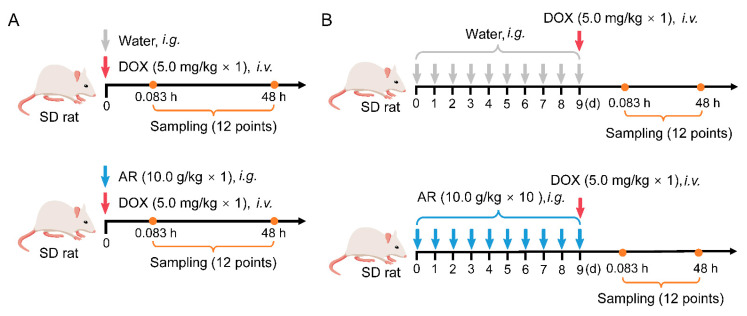
Schematic of animal experiments. (**A**) Mice received 5.0 mg/kg DOX via the tail vein after a single dose of 10 g/kg AR administration. (**B**) Mice received 5.0 mg/kg DOX via the tail vein after 10 days pre-treatment with AR (10 g/kg/d). DOX: doxorubicin, AR: Astragali Radix.

**Figure 2:**
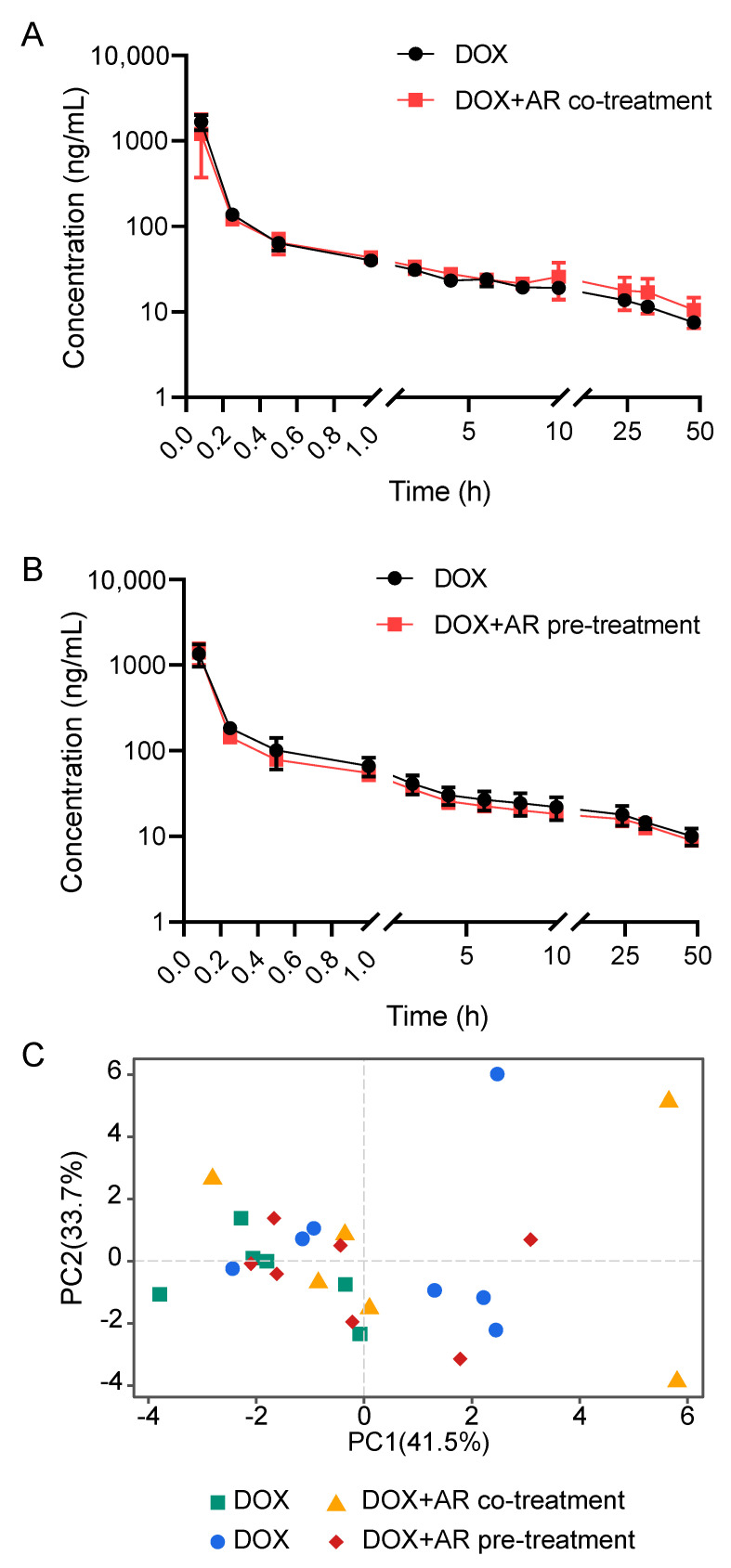
Astragali Radix (AR) does not affect the plasma pharmacokinetics of doxorubicin (DOX). (**A**) Mean plasma concentration–time curves of DOX after intravenous administration of DOX (5 mg/kg) alone and the co-treatment with AR (10 g/kg). (**B**) Mean plasma concentration–time curves of DOX after intravenous administration of DOX (5 mg/kg) alone and the pre-treatment with AR (10 g/kg × 10). (**C**) The principal component analysis score plot shows no clear separation among four groups of rats. All data are presented as mean ± SD.

The current LC-MS/MS method was successfully applied for investigating the plasma pharmacokinetics and tissue distribution of DOX in rats after co-treatment with single-dose AR or pre-treatment with multiple-dose AR (Figure 1). The mean plasma concentration–time curves of DOX are shown in Figure 2A,B, and all 14 pharmacokinetic parameters are summarized in Table 3. For the two isotype control groups that received only DOX, the pharmacokinetic parameters of the maximum experimental concentration (C_max_), the total area under the concentration–time curve (AUC_0-t_), and the half-life (t_1/2_) were in the range of 838.4 ng/mL to 2157.8 ng/mL, 1054.7 µg/L*h to 1940.1 µg/L*h, and 25.0 h to 39.3 h, respectively. These results were consistent with previously reported animal experiment data [19,20]. Compared with the DOX groups, we found no significant differences in all pharmacokinetic parameters (e.g., C_max_, AUC_0-t_, t_1/2_, and MRT) after AR co- or pre-treatment. Furthermore, we explored the variation patterns of these 14 pharmacokinetic parameters among the four groups of rats using principal component analysis (PCA). As illustrated in Figure 2C, no clear separation is observed between the four groups, although there are two discrete samples. These results indicate that AR co- or pre-treatment could not change the drug disposition of DOX in vivo.

**Figure 3:**
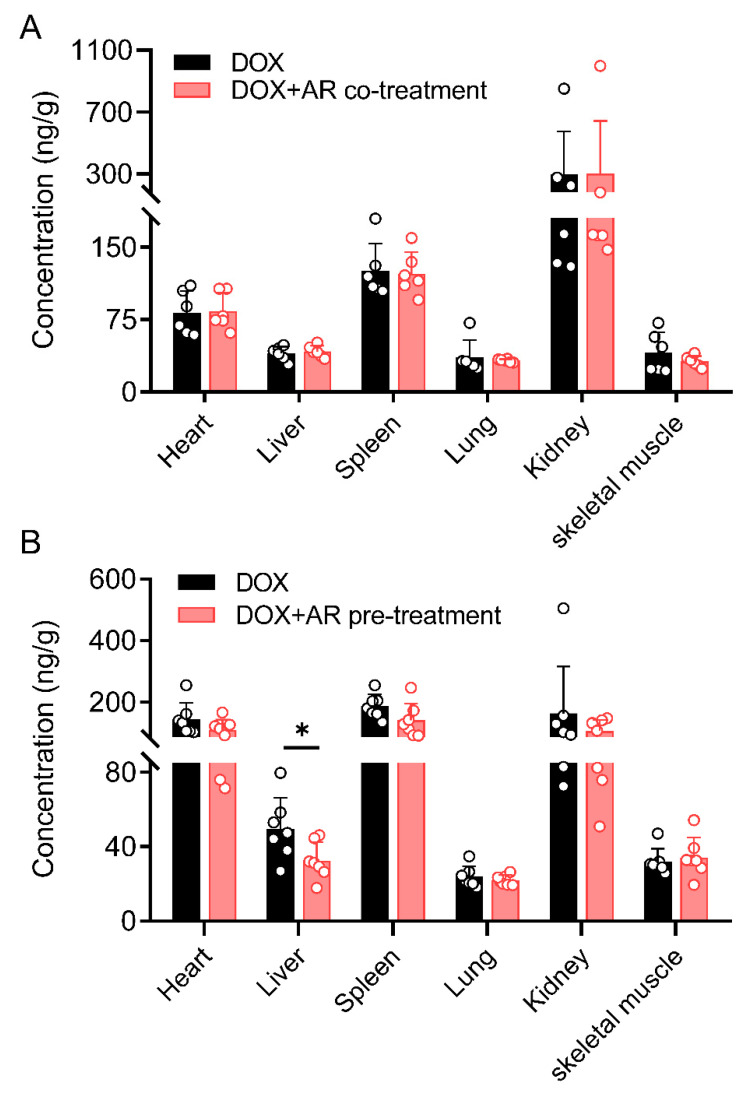
Astragali Radix (AR) has little effect on the tissue distribution of doxorubicin (DOX). (**A**) Tissue distribution of DOX at 48 h after intravenous injection of DOX (5 mg/kg) alone and the co-treatment with AR (10 g/kg). (**B**) Tissue distribution of DOX at 48 h after intravenous injection of DOX (5 mg/kg) alone and the pre-treatment with AR (10 g/kg × 10). All data are presented as mean ± SD. Student *t*-test, * *p* < 0.05.

At 48 h after injection, DOX concentrations in various tissues, namely heart, liver, lung, kidney, spleen, and skeletal muscle, were detected at levels above the LOQ. Extensive distribution was seen in some organs, such as the kidney, spleen, and heart, whereas relatively low levels were detected in the lung and skeletal muscle (Supplementary Table S3), which was in accordance with the known disposition property of DOX [21]. As seen in Figure 3, the concentrations of DOX in these tissues were not affected by the co-treatment with a single dose of AR. In contrast, the pre-treatment with multiple doses of AR reduced DOX tissue exposure but only showed statistical significance (*p* < 0.05) in the liver. These data suggest that the short-term (≤10 d) administration of AR appears not to change the tissue distribution of DOX.

### 2.3. Network-Based Measures of Doxorubicin–Astragali Radix Relationship

**Figure 4:**
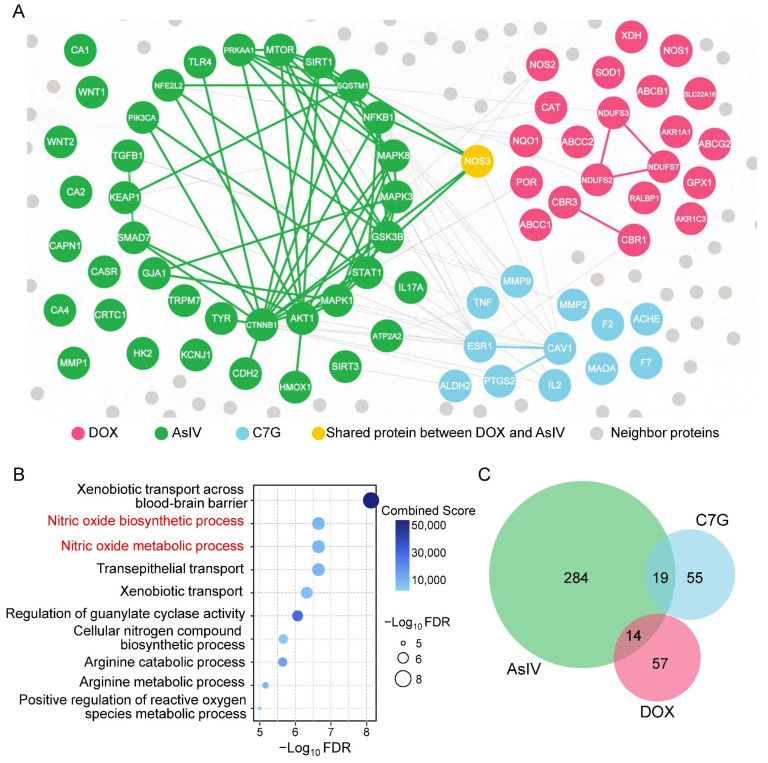
Network-based relationship between Astragali Radix (AR) components and doxorubicin (DOX) disposition. (**A**) A subnetwork of the protein–protein interactome illustrating the network-based separation between DOX, Astragaloside IV (ASIV), and calycosin-7-O-glucoside (C7G). (**B**) Top 10 enriched pathways related to DOX disposition. (**C**) Venn diagram showing little overlap between significantly (FDR < 0.01) enriched pathways of DOX, ASIV, and C7G.

To understand why AR failed to change the plasma pharmacokinetics and tissue distribution of DOX, we turned to quantifying the network-based relationship between the modules related to DOX disposition and AR components. We found that five of the nine representative components of AR were not localized to the vicinity of the DOX disposition-associated module, of which the network-based distances (*S_AB_*) values were greater than 0 (Supplementary Table S4). For example, Astragaloside IV (AsIV) and calycosin-7-O-glucoside (C7G), two quality control markers of AR specified by Chinese Pharmacopoeia [22], were topologically separated from DOX with *S_AB_* values of 0.059 and 0.085, respectively (Figure 4A). Pathway enrichment analysis further confirmed the results of the network-based evaluation. It was revealed that 22 enzymes involved in DOX transport and metabolism were significantly enriched in the nitric oxide-related pathways (Figure 4B), while the enriched pathways of AsIV and C7G had few overlaps with those of DOX (Figure 4C). Altogether, it is not easy for AR to directly interfere with the DOX disposition in vivo because of their topological separation.

## 3. Discussion

The combination of herbal medicine and chemotherapy offers widespread, well-documented advantages in treating cancer [23]. The main objective of this study was to uncover the effects of co- and pre-treatment of AR on the plasma pharmacokinetics and tissue distribution of DOX in rats. To achieve this goal, we first developed an LC-MS/MS analytical method for the reliable quantification of DOX in different biological matrices, including rat plasma and six types of tissues. The proposed method enabled the accomplishment of easy sample preparation (one-step protein precipitation), high sensitivity (LOQ < 5 ng/mL or 20 ng/g), good repeatability (RSD < 15%), and wide dynamic range (up to three orders of magnitude). Compared with recently reported methods developed based on the UPLC system [24,25], our method, which used HPLC, considerably reduced instrument requirements while providing acceptable throughput (11 min/run). Moreover, the method was fully validated in each of the seven biological matrices, following the validation criteria established by the FDA. The validation results not only demonstrate the robustness of the LC-MS/MS method but also guarantee sufficient sensitivity and specificity for the reliable quantification of DOX present in different biological samples, as well as at different time points.

DOX, in clinics, is administered as a single high dose, frequent low doses, or a continuous infusion [1]. Here, we determined the concentrations of DOX in rat plasma and six tissues following a single intravenous dose of 5 mg/kg. Consistent with previous studies [12,26], our data show that the intravenous bolus injection of DOX produces high plasma concentrations, which fall quickly due to a rapid and extensive distribution into tissues (Figure 3). More importantly, we found that short-term (≤10 d) administration of AR, whether a single-dose co-treatment or multiple-dose pre-treatment, was ineffective at changing the plasma pharmacokinetics of DOX (Table 3). Meanwhile, the tissue exposure of DOX seemed to be slightly affected by AR pre-treatment, showing a downward trend, but only a significant difference was observed in the liver (Figure 3). The network-based measures revealed the unexpected weak overlapping between nine representative AR components and DOX disposition. For example, there was only one shared node (NOS3) between the network modules of AsIV and DOX (Figure 4). Previous network medicine studies have demonstrated that, for a drug to have a therapeutic effect, the drug-target module must overlap with the disease module [18]. Thus, our findings suggest that AR components are unlikely to act on the enzymes that metabolize or transport DOX. In other words, AR may mitigate DOX-induced toxicity by affecting drug targets rather than drug disposition. Furthermore, AR is not only a herbal medicine but also a legal dietary supplement in both China and the USA [27]. People, such as elderly adults who feel weak, may take AR as a health product on a daily basis. Future work is needed to explore whether the effects of AR on DOX disposition become pronounced under long-term (months or even years) treatment.

Our study has several strengths. We used a clinically relevant regimen of low DOX and AR doses to establish the animal model. We used an LC-MS/MS method that was well-validated and possessed good quantitative capability and reproducibility. The network-based approach contributed to identifying mechanisms of the combination usage of DOX and AR. However, there are several limitations to the current analysis. Some in vivo metabolites of DOX (i.e., doxorubicinol) are cytotoxic, while others (i.e., doxorubicinone) are not [28]. Although AR shows a weak effect on the concentrations of DOX in rat plasma and tissues, the production of each metabolite of DOX may vary. To increase the coverage of analytes, we may update the LC-MS/MS method to further determine both DOX and its primary metabolites simultaneously. Although DOX levels in each tissue were successfully quantified at 48 h after intravenous administration, adding two to three sampling time points, such as at 0.5 h, 1 h, and 4 h, may be more appropriate to fully characterize the tissue distribution of DOX, as well as the effects of AR. In addition, owing to the complex combination of DOX and AR dosing regimens, further experiments on the multiple doses and long-term administration are warranted.

## 4. Materials and Methods

### 4.1. Chemicals and Reagents

Doxorubicin hydrochloride injection was obtained from Shenzhen Main Luck Pharmaceuticals Inc. (Shenzhen, China). Astragali Radix crude slices were purchased from Gansu Longmaotong Chinese Herbal Medicine Trading Co., Ltd. (Lanzhou, China). Chemical standards, including DOX and daunorubicin, were purchased from Shanghai Yuanye Biotechnology Co., Ltd. (Shanghai, China). LC-MS-grade methanol was bought from Merck KGaA (Darmstadt, Germany) and HPLC-grade formic acid was obtained from ROE Scientific Inc. (Newark, DE, USA). Water was purified with a Milli-Q system (Millipore Corporation, Bedford, MA, USA). Isoflurane, a general inhalation anesthetic, was purchased from Shenzhen Reward Life Technology Co., Ltd. (Shenzhen, China) and heparin sodium was purchased from Shanghai Aladdin Bio-Chem Technology Co., Ltd. (Shanghai, China).

### 4.2. Preparation of Astragali Radix Water Extract

The AR water extract was prepared by the procedure in our previous study [9]. Briefly, AR crude slides were accurately weighed and boiled twice in deionized water (1:10, *w*/*v*) for 2 h each time. The two filtrates were pooled together and then lyophilized to obtain a freeze-dried powder. To ensure the quality of the AR extract, the contents of 9 components in the powder were determined, including Astragaloside I, Astragaloside II, Astragaloside III, Astragaloside IV, Isoastragaloside II, calycosin, calycosin-7-O-glucoside, formononetin, and ononin (Supplementary Methods).

### 4.3. Sample Preparation

Frozen tissue samples were thawed at 4 °C. Each 100 mg of tissue was added with 400 μL of pre-cold saline, except for the kidney sample, to which 1000 µL of saline was added. Then, tissues were homogenized seven times (6.5 m/s for 10 s) with 30 s intervals between the homogenization steps. Tubes were centrifuged at 6000× *g* for 15 min at 4 °C, and supernatants were transferred to clean tubes. A 50 μL aliquot of plasma or tissue homogenate was added with 10 μL of internal standard (IS) solution (daunorubicin, 500 ng/mL) and 200 μL of methanol. The mixture was thoroughly vortexed for 5 min and centrifugated twice at 19,000× *g* for 10 min at 4 °C. The supernatant was transferred for further LC-MS/MS analysis.

### 4.4. LC-MS/MS Analysis

Chromatographic separation was performed in a Nexera UPLC system (Shimadzu, Japan) equipped with a ZORBAX SB-C18 column (150 mm × 2.1 mm, 5 μm, Agilent, Santa Clara, CA, USA). The sample injection volume was 5 µL. The mobile phase consisted of 0.1% formic acid in water (A) and methanol (B) with a 0.3 mL/min flow rate. An 11 min elution gradient was performed as follows: the proportion of B was set at 60% for the first 1 min, increased linearly to 95% in the next 2.5 min, and then maintained for 2 min; finally, the initial conditions were restored within 1.5 min and kept for 4 min for column conditioning. The temperatures of the column and autosampler were set at 30 °C and 4 °C, respectively.

MS detection was performed using a Triple Quad^TM^ 8040 system (Shimadzu, Japan) equipped with an electrospray ionization (ESI) source working in the positive-ion mode. The optimized multiple reaction monitoring (MRM) transitions of DOX and IS were m/z 544.1 → 397.1 and m/z 528.1 → 320.9, respectively. Collision energy (CE) was set at 12 V for DOX and 29 V for IS. The key instrument parameters were set as follows: capillary voltage, 4.5 kV; nebulizing gas, 3 L/min; drying gas, 15 L/min; heat block temperature, 400 °C; desolvation temperature, 250 °C. Raw data were obtained and processed using LabSolutions LCMS software 5.86 (Shimadzu, Japan).

### 4.5. Method Validation

The LC-MS/MS method was developed following the compliance criteria described by the FDA Bioanalytical Method Validation Guidance for Industry, in terms of lower limit of detection (LLOD), lower limit of quantification (LLOQ), linearity, precision, accuracy, recovery, matrix effect, and stability.

Working solutions were spiked with blank plasma or tissue homogenate to yield a calibration curve. The 8-point calibration curve was constructed by plotting the peak area ratios of DOX to the IS against the nominal concentrations of DOX. Each calibration curve was statistically analyzed using weighted least squares. LLOD and LLOQ were calculated at a signal-to-noise ratio (S/N) of at least 3 and 10, respectively. The inter-day and intra-day accuracy and precision were evaluated by analyzing four (LLOQ, low, medium, and high) levels of quality control (QC) samples on three consecutive days, and each level contained five replicates on the same day. Precision was expressed by the relative standard deviation (RSD), with the acceptance criteria of less than 20% for the LLOQ and 15% for the other concentrations. Accuracy was calculated by the percentage ratio of measured concentrations to the nominal value, with the acceptance criteria of 80–120% for the LLOQ and 85–115% for the other concentrations.

A recovery experiment was carried out at three (low, medium, and high) levels of QCs, with five replicates at each level. Recovery was calculated by comparing the analytical results of extracted samples with corresponding extracts of blanks spiked with the analyte post-extraction. Matrix effect (ME) was evaluated at two (low and high) QC levels and calculated by comparing the peak areas of DOX and IS spiked into the post-extracted biological matrix with the solution containing the equivalent amounts of analytes. There was no matrix effect if the ratio was in the range of 85–115%. The stability of DOX was assessed in terms of autosampler (4 °C for 12 h), benchtop (room temperature for 10 h), three freeze-thaw cycles, and long-term (−80 °C for 30 d). Stability QCs were compared against freshly prepared QCs and considered stable if the accuracy was within 85–115% and RSD ≤ 15%.

### 4.6. Pharmacokinetics and Tissue Distribution Studies

Male Sprague-Dawley (SD) rats (220 ± 20 g) were purchased from Shanghai Sippr-BK Lab Animal Co., Ltd. (Shanghai, China). Animals were housed in a standard laboratory condition with controlled temperature (25 °C), humidity (45 ± 5%), and dark/light cycle (12/12 h). All experimental procedures were conducted according to the guidelines for the care and use of laboratory animals and approved by the Animal Ethics Committee of China Pharmaceutical University.

To uncover the effects of single-dose AR administration on DOX pharmacokinetic behavior and tissue distribution, 12 rats were randomly divided into two groups (*n* = 6 per group): DOX and DOX+AR co-treatment groups. Animals in the DOX+AR co-treatment group received a single dose of 5.0 mg/kg DOX via the tail vein immediately after 10 g/kg AR intragastrical administration. In contrast, the DOX group was given the same dose of DOX and an equal volume of water. An additional 14 rats were employed to investigate the effects of multiple-dose AR pre-treatment. Animals were randomly divided into two groups (*n* = 7 per group) that were given a single dose of DOX (5.0 mg/kg, i.v.) after 10 days of pre-treatment with AR (10 g/kg/d, i.g.) or an identical volume of water.

Approximately 0.2 mL of blood samples were collected from the jugular vein catheter, at 0, 0.083, 0.25, 0.5, 1, 2, 4, 6, 8, 10, 24, 32, and 48 h, into centrifuge tubes containing heparin. Plasma samples were obtained after centrifugation (2000× *g* for 10 min at 4 °C). A corresponding volume of saline was supplemented after each blood collection. All rats resumed eating 4 h after DOX administration and were free to move around and drink during the pharmacokinetic experiment. The rats were sacrificed by anesthesia at 48 h after DOX administration. Tissue samples, including heart, liver, lung, kidney, spleen, and skeletal muscle, were harvested. All plasma and tissue samples were stored at −80 °C for further analysis.

Pharmacokinetic parameters were calculated using DAS software 3.2.8 (Mathematical Pharmacology Professional Committee of China, China) with the noncompartmental method. Differences in continuous variables were compared using Student’s *t*-test. *p* < 0.05 was considered significant.

### 4.7. Network Analysis

To evaluate the network-based relationship of DOX disposition (A) and an AR component (B), we used a recently introduced separation measure [29]:

$$S_{AB}\equiv \langle d_{AB}\rangle -\frac{\langle d_{AA}\rangle +\langle d_{BB}\rangle }{2}$$

where $\langle d_{AA}\rangle$ and $\langle d_{BB}\rangle$ are the shortest distances between proteins within the network modules of DOX metabolism and an AR component, respectively; $\langle d_{AB}\rangle$ is the shortest distance between A-B protein pairs. For *S_AB_* < 0, the proteins of DOX and an AR component are located in the same network neighborhood, while for *S_AB_* ≥ 0, the two drug proteins are topologically separated.

A state-of-the-art human PPI, including 18,198 unique proteins and 271,278 edges, was used as the background network in this study [30]. We collected 22 enzymes that play a role in DOX transport and metabolism from PharmGKB (www.pharmgkb.org/, accessed on 9 June 2022) and selected 9 quantifiable components as representatives of AR, whose targets had been reported in our previous work (Supplementary Data) [9]. The network analysis was performed using Mathematica software (version 13.0, Wolfram Research, USA).

## 5. Conclusions

In summary, we reported the effects of AR on the pharmacokinetics and tissue distribution of DOX using a novel analytical method to determine DOX in rat plasma and six tissues. Specifically, we observed that the plasma pharmacokinetics of DOX was not affected by AR co- and pre-treatment, whereas DOX concentrations in six tissues were slightly decreased under AR pre-administration for 10 consecutive days. In addition, network-based measures revealed that representative AR components could hardly act on the enzymes involved in DOX metabolism and transport. This study provided valuable data for understanding the DOX–AR combination and might help uncover the mechanisms of AR in alleviating DOX-induced toxicity.

## Figures and Tables

**Table 1:** The linear regression parameters for doxorubicin in rat plasma and six tissues.

Biological Matrix	Linear Range	Calibration Curve	r^2^	LLOQ
Plasma	5–5000 ng/mL	Y = 0.0116263X + 0.0132798	0.9993	5 ng/mL
Liver	20–2400 ng/g	Y = 0.00274442X + 0.00861329	0.9988	20 ng/g
Heart	20–2400 ng/g	Y = 0.00283048X + 0.0101572	0.9975	20 ng/g
Kidney	50–6000 ng/g	Y = 0.00107142X + 0.00120827	0.9978	50 ng/g
Spleen	20–2400 ng/g	Y = 0.00244706X − 0.00562543	0.9950	20 ng/g
Lung	20–2400 ng/g	Y = 0.00276342X + 0.00405469	0.9979	20 ng/g
Skeletal muscle	20–2400 ng/g	Y = 0.00282242X + 0.0109277	0.9977	20 ng/g

**Table 2:** The precision and accuracy for doxorubicin in rat plasma and six tissues.

Biological Matrix	Concentration	Intra-Day (*n* = 5)		Inter-Day (*n* = 15)	
		Accuracy (%)	Precision (RSD%)	Accuracy (%)	Precision (RSD%)
Plasma	5 ng/mL	96.08	6.44	97.12	5.46
	10 ng/mL	94.82	6.12	95.70	6.53
	500 ng/mL	100.59	4.30	101.79	5.48
	4000 ng/mL	98.15	3.54	100.75	6.13
Liver	20 ng/g	95.66	6.22	97.25	5.85
	40 ng/g	90.33	4.56	93.10	5.45
	400 ng/g	92.23	6.50	95.75	5.67
	2000 ng/g	93.88	4.05	98.00	5.89
Heart	20 ng/g	99.82	6.85	95.99	8.79
	40 ng/g	103.21	4.28	100.30	5.15
	400 ng/g	96.43	3.47	100.41	5.54
	2000 ng/g	104.26	5.39	100.07	6.06
Spleen	20 ng/g	96.44	6.36	97.96	6.39
	40 ng/g	91.86	5.61	95.55	6.06
	400 ng/g	89.15	2.11	95.04	7.75
	2000 ng/g	92.44	4.49	96.44	5.98
Kidney	50 ng/g	99.50	5.46	98.78	5.90
	100 ng/g	98.54	6.05	95.03	7.72
	1000 ng/g	102.07	5.90	94.98	7.68
	5000 ng/g	100.59	4.86	96.87	6.11
Lung	20 ng/g	90.87	10.16	95.79	9.23
	40 ng/g	91.56	2.22	99.08	7.90
	400 ng/g	99.31	6.76	97.93	5.50
	2000 ng/g	103.38	4.95	101.80	4.40
Skeletal muscle	20 ng/g	96.81	9.01	97.02	8.55
	40 ng/g	91.89	1.17	92.36	3.78
	400 ng/g	95.22	3.69	99.25	5.23
	2000 ng/g	98.20	4.39	100.40	4.93

**Table 3:** The pharmacokinetic parameters after intravenous administration of 5 mg/kg doxorubicin (DOX) following the treatment with Astragali Radix (AR) (mean ± SD).

Parameters ^#^	Unit	AR Co-Treatment (*n* = 6 per Group)			AR Pre-Treatment (*n* = 7 per Group)		
		DOX	DOX + AR (10g/kg × 1)	*p*-Value ^$^	DOX	DOX + AR (10g/kg × 10)	*p*-Value ^$^
AUC_(0-t)_	μg/L*h	1212.08 ± 107.82	1265.88 ± 226.98	0.642	1303.35 ± 271.74	1208.74 ± 145.35	0.467
AUC_(0-∞)_	μg/L*h	1561.04 ± 147.02	1773.14 ± 288.55	0.174	1763.3 ± 339.93	1626.07 ± 231.54	0.430
AUMC_(0-t)_	h*h*μg/L	13,438.17 ± 881.77	18,109.93 ± 6093.09	0.148	17,173.75 ± 3436.44	15,248.33 ± 1891.35	0.252
AUMC_(0-∞)_	h*h*μg/L	46,004.25 ± 8764.93	67427.17 ± 25,880.54	0.129	60,856.88 ± 14,612.33	55,604.05 ± 15,045.69	0.551
MRT_(0-t)_	h	11.13 ± 0.74	14.38 ± 3.67	0.105	13.24 ± 1.11	12.69 ± 1.51	0.486
MRT_(0-∞)_	h	29.34 ± 4.25	37.7 ± 11.6	0.161	34.59 ± 6.48	33.81 ± 5.55	0.826
VRT_(0-t)_	h^2	197.67 ± 4.35	209.5 ± 15.52	0.154	212.17 ± 6.72	211.82 ± 7.21	0.933
VRT_(0-∞)_	h^2	1766.66 ± 538.86	2213.68 ± 1222.38	0.472	2022.61 ± 650.14	2067.43 ± 786.43	0.916
λz	1/h	0.023 ± 0.004	0.022 ± 0.004	0.621	0.022 ± 0.003	0.023 ± 0.005	0.830
C_last	μg/L	7.86 ± 0.8	10.85 ± 3.98	0.156	10.04 ± 2.17	8.99 ± 1.02	0.307
t_1/2_	h	30.52 ± 4.47	32.59 ± 7.48	0.607	31.79 ± 5.12	32.05 ± 6.95	0.944
V	L/kg	141.38 ± 18.15	135.98 ± 35.78	0.770	133.85 ± 28.5	142.17 ± 25.7	0.605
CL	L/h/kg	3.23 ± 0.33	2.89 ± 0.43	0.189	2.92 ± 0.43	3.13 ± 0.38	0.390
C_max_	μg/L	1667.94 ± 304.04	1211.41 ± 764.75	0.243	1351.21 ± 364.86	1411.01 ± 368.38	0.782

^#^ Abbreviations: AUC, area under the concentration–time curve; AUMC, area under the first moment curve; MRT, mean residence time; VRT, variance of mean residence time; C_last, the predicted last concentration; CL, plasma clearance; V, apparent volume of distribution; Cmax, maximum plasma concentration. ^$^ Student *t*-test.

## Data Availability

Data is contained within the article and Supplementary Material.

## Supplementary Materials

The following supporting information can be downloaded at: https://www.mdpi.com/article/10.3390/ph15091104/s1. Figure S1: The representative extracted ion chromatograms of DOX and IS in rat plasma and tissues. Table S1: The recovery and matrix effect for DOX and IS in rat plasma and six tissues. Table S2: The stability of doxorubicin under various storage conditions. Table S3: The concentrations of DOX in six rat tissues at 48 h after intravenous administration of 5 mg/kg DOX. Table S4: Network-based separation between nine AR components and DOX disposition.
