# Supplementary material for: Plasma Pharmacokinetics and Tissue Distribution of Doxorubicin in Rats following Treatment with Astragali Radix
Source: Pharmaceuticals (Basel). 2022 Sep 4;15(9):1104. doi: 10.3390/ph15091104 (PMC9505068; doi:10.3390/ph15091104)
Supplement: Supplementary file 1 [file pharmaceuticals-15-01104-s001.zip › pharmaceuticals-1879782-supplementary.pdf]

Supplementary Information

Plasma pharmacokinetics and tissue distribution of doxorubicin in rats following treatment with Astragali Radix

Yin Huang[a,b], Fang Yang[b], Linling Guo[b], Yan Xu[b], Xiaxia Yu[c], Zunjian Zhang[b*], Yuxin Zhang[a*]

[a] China Pharmaceutical University Nanjing Drum Tower Hospital, Nanjing, 210009, China

[b] Key Laboratory of Drug Quality Control and Pharmacovigilance, China Pharmaceutical University, Ministry of Education, Nanjing, 210009, China

[c] Department of Pharmacy, Affiliated Zhongda Hospital, School of Medicine, Southeast University, Nanjing 210009, China

*Corresponding author:

Zunjian Zhang, Ph.D.

Key Laboratory of Drug Quality Control and Pharmacovigilance

China Pharmaceutical University, Ministry of Education

Email: zzj@cpu.edu.cn

Yuxin Zhang, Ph.D.

China Pharmaceutical University Nanjing Drum Tower Hospital

Email: juliandth@163.com

Methods

Quality control of Astragali Radix water extract

Sample preparation

The freeze-dried powder containing approximately 100 mg crude slices was added with 1,000 μL of methanol/water (70:30, V/V), and vortexed for 1 min. The mixture was then extracted ultrasonically for 10 min, and centrifuged for 10 min at 14,000 rpm. The supernatant was diluted 10 times with methanol/water (70:30, V/V) and filtered through a 0.22 μm filter. Finally, 80 μL of supernatant was added with 20 μL of digoxin (50 μg/mL, internal standard), vortexed for 1 min, and centrifuged at 14000 rpm for 10 min. The supernatant was transferred for LC-MS/MS analysis.

LC-MS/MS analysis

The quantification of 9 representative components of Astragali Radix (AR) was achieved using a Shimadzu Nexera UFLC system coupled to a MS-8040 triple quadrupole mass spectrometer system (Japan). The separation was performed on a Zorbax SB-C18 (2.1×100 mm,1.8 μm) column with a flow rate of 0.30 mL/min at 40°C. The mobile phase A was 0.1% formic acid in water, and mobile B was acetonitrile. The gradient condition for separation was optimized as follows: 0-2 min, 10-35% B; 2-7 min, 35% B; 7-15 min, 35-90% B; 15-16 min, 90% B; 16-17 min, 90-10% B; 17-20 min, 10% B. The electrospray ionization (ESI) source was operated in the positive mode with multiple reaction monitoring (MRM). The main instrument parameters were as follows: nebulizing gas, 3 L/min; drying gas, 15 L/min; spray voltage, -3.5 KV; heat block temperature, 400°C; desorption line temperature, 250°C. The optimized MRM parameters and the quantification results are given in the following table.

No.	Analytes	MW	Precursor ion (m/z)	Product ion (m/z)	CE (eV)	Content (μg/g crude slices)
1	Astragaloside I	869.04	870.1	143.2	-19	88.27
2	Astragaloside II	827.0	827.5	143.2	-17	136.83
3	Astragaloside III	784.97	786.1	143.2	-15	13.13
4	Astragaloside IV	784.97	786.1	143.2	-15	59.93
5	Isoastragaloside II	827.0	827.5	143.2	-17	66.67
6	Calycosin	284.28	285.1	270.0	-24	102.53

7	Calycosin-7-O-glucoside	446.44	446.8	285.0	-18	437.33
8	Formononetin	268.28	269.1	197.05	-41	50.47
9	Ononin	430.44	431.1	269.0	-17	169.23
IS	Digoxin	780.9	781.5	781.5	-5	

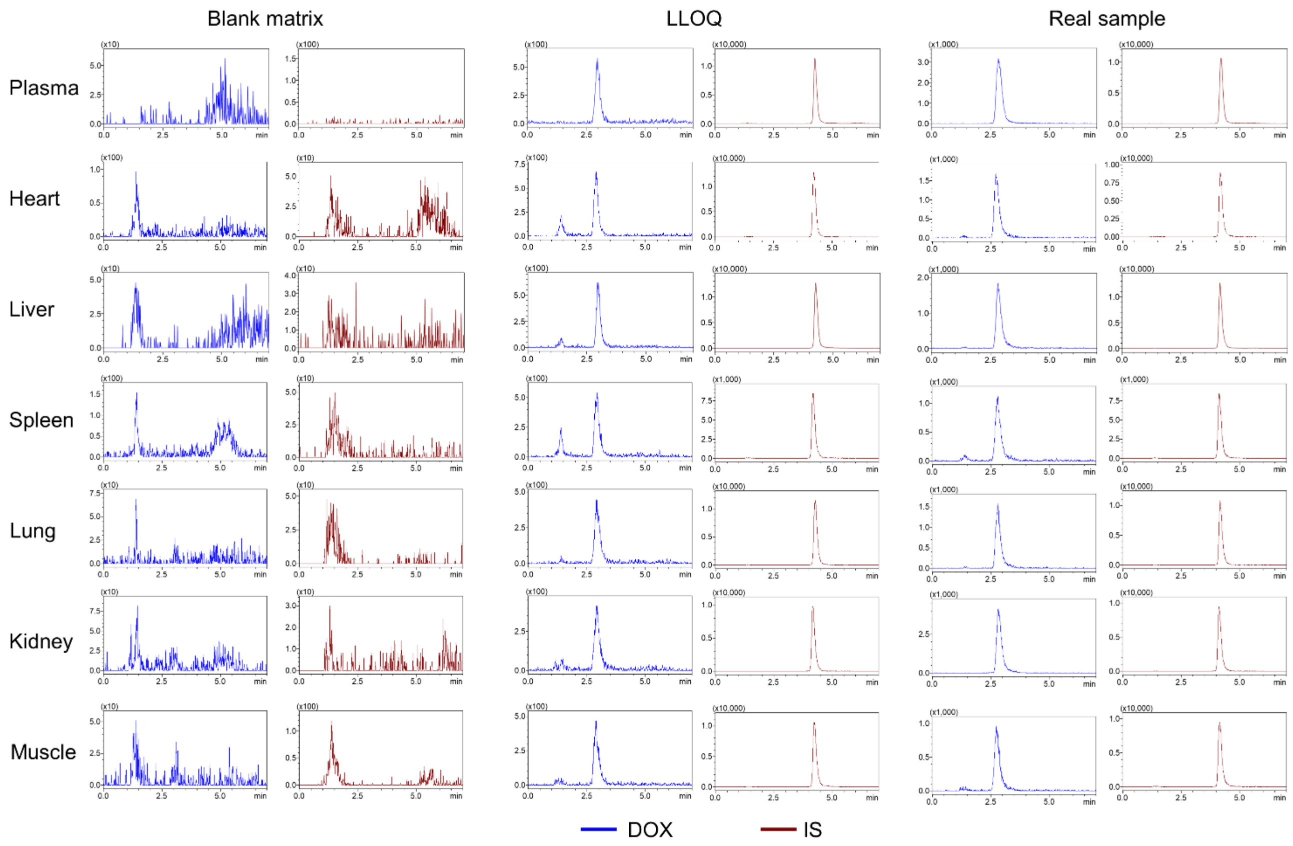

Figure S1 The representative extracted ion chromatograms of DOX and IS in rat plasma and tissues. Retention times of DOX and IS were 2.85 min and 4.23 min, respectively.

Table S1-1 The recovery and matrix effect for doxorubicin in rat plasma and six tissues.

Biological matrix	Concentration	Recovery (n=5)		Matrix effect (n= 6)	
		Mean (%)	RSD (%)	Mean (%)	RSD (%)
Plasma	10 ng/mL	95.49	6.56	97.40	7.35
	500 ng/mL	92.94	1.26		
	4000 ng/mL	87.34	2.74	89.71	2.28
Liver	40 ng/g	92.93	2.10	99.66	5.03
	400 ng/g	88.40	4.11		
	2000 ng/g	92.69	4.47	89.8	1.54
Heart	40 ng/g	99.33	6.80	95.34	5.94
	400 ng/g	90.98	7.35		
	2000 ng/g	85.05	3.27	91.57	4.34
Spleen	40 ng/g	72.23	5.16	93.38	5.36
	400 ng/g	67.98	2.21		
	2000 ng/g	66.34	6.74	94.57	2.53
Kidney	100 ng/g	77.32	5.10	90.92	4.75
	1000 ng/g	80.62	3.58		
	5000 ng/g	78.64	5.76	87.37	1.98
Lung	40 ng/g	87.76	3.82	90.12	5.37
	400 ng/g	82.98	2.55		
	2000 ng/g	87.62	3.32	88.19	0.94
Skeletal muscle	40 ng/g	93.48	1.89	88.44	1.31
	400 ng/g	88.84	1.99		
	2000 ng/g	94.07	4.85	89.15	6.38

Table S1-2 The recovery and matrix effect for IS in rat plasma and six tissues.

Biological matrix	Concentration (ng/mL)	Recovery (n=5)		Matrix effect (n= 6)	
		Mean	RSD	Mean	RSD

		(%)	(%)	(%)	(%)
Plasma	500	95.89	2.48	100.67	8.71
Liver	500	98.24	4.30	99.04	2.65
Heart	500	96.27	2.75	99.29	6.16
Spleen	500	90.77	3.87	98.88	6.62
Kidney	500	86.58	2.93	89.07	2.87
Lung	500	85.95	1.30	99.38	2.50
Skeletal muscle	500	92.25	2.98	96.95	4.28

Table S2 The stability of doxorubicin under various storage conditions (n = 3).

Biological matrix	Concentration	Short-term (room temperature, 10 h)		Auto-sampler (4 °C, 12 h)		Three freeze-thaw cycles (-80 °C)		Long-term (-80 °C for 30 d)	
		Mean (%)	RSD (%)	Mean (%)	RSD (%)	Mean (%)	RSD (%)	Mean (%)	RSD (%)
Plasma	10 ng/mL	92.06	10.22	100.72	4.42	88.62	11.27	108.29	0.30
	500 ng/mL	102.22	0.65	92.17	4.97	103.25	7.25	93.66	3.36
	4000 ng/mL	103.47	5.52	90.45	2.35	101.04	7.03	94.93	4.08
Liver	40 ng/g	94.47	1.51	97.64	5.27	109.06	1.21	106.67	4.14
	2000 ng/g	87.30	1.72	92.66	2.19	102.36	1.21	106.76	1.46
Heart	40 ng/g	91.00	4.90	112.70	2.10	96.65	7.99	99.06	4.81
	2000 ng/g	87.73	1.33	98.93	2.96	89.13	1.39	95.28	4.76
Spleen	40 ng/g	94.10	1.24	107.40	5.25	101.67	4.36	103.25	3.95
	2000 ng/g	100.07	1.91	111.77	2.64	107.61	3.09	108.42	2.59
Kidney	100 ng/g	101.12	1.04	102.62	3.97	102.67	2.45	101.34	1.62
	5000 ng/g	100.51	2.16	101.27	3.60	104.11	3.00	103.90	11.43
Lung	40 ng/g	93.08	6.77	102.87	4.37	98.11	5.16	98.53	0.87
	2000 ng/g	95.45	5.40	95.14	4.58	107.42	2.38	107.07	1.98
Skeletal muscle	40 ng/g	101.93	1.35	105.95	4.20	94.27	1.23	104.41	1.88
	2000 ng/g	95.26	2.09	101.27	5.08	102.73	1.95	90.79	5.41

Table S3 The concentrations of DOX in six rat tissues at 48 h after intravenous administration of 5 mg/kg DOX (mean ± SD).

Tissue	AR co-treatment		AR pre-treatment	
	DOX	DOX+AR (10g/kg x 1)	DOX	DOX+AR (10g/kg x 10)
Liver	40.07 ± 6.49	41.91 ± 5.47	49.53 ± 15.84	32.41 ± 9.26*
Heart	82.21 ± 20.34	83.76 ± 17.26	142.54 ± 50.06	108.91 ± 30.40
Spleen	125.17 ± 25.89	122.66 ± 20.19	185.60 ± 36.17	139.94 ± 50.68
Kidney	296.65 ± 253.30	302.02 ± 310.97	162.41 ± 142.05	104.66 ± 33.78
Lung	36.25 ± 15.96	32.64 ± 1.43	23.64 ± 5.23	21.89 ± 2.46
Skeletal muscle	40.84 ± 19.15	31.65 ± 5.25	31.93 ± 6.36	34.11 ± 9.87

Table S4 Network-based separation between nine AR components and DOX disposition.

Component	PubChem ID	Target number	S_{AB}
Astragaloside III	441905	6	0.168
Isoastragaloside II	60148655	2	0.148
Calycosin-7-O-glucoside	5318267	12	0.085
Ononin	442813	14	0.074
Astragaloside IV	13943297	39	0.059
Formononetin	5280378	62	-0.006
Calycosin	5280448	41	-0.130
Astragaloside I	51346122	9	-0.242
Astragaloside II	11968895	14	-0.282
